# Disease severity–related alterations of cardiac microRNAs in experimental pulmonary hypertension

**DOI:** 10.1111/jcmm.15352

**Published:** 2020-05-12

**Authors:** Zuzana Kmecova, Jana Veteskova, Katarina Lelkova‐Zirova, Lenka Bies Pivackova, Gabriel Doka, Eva Malikova, Ludovit Paulis, Peter Krenek, Jan Klimas

**Affiliations:** ^1^ Department of Pharmacology and Toxicology Faculty of Pharmacy Comenius University Bratislava Slovakia; ^2^ Institute of Pathophysiology Faculty of Medicine Comenius University Bratislava Slovakia; ^3^ Institute of Normal and Pathological Physiology Centre of Experimental Medicine Slovak Academy of Sciences Bratislava Slovakia

**Keywords:** Fulton index, microRNA, miR‐1, miR‐133a, miR‐208a, miR‐214, miR‐499, monocrotaline, pulmonary hypertension

## Abstract

Right ventricular (RV) failure is the primary cause of death in pulmonary arterial hypertension (PAH). We hypothesized that heart‐relevant microRNAs, that is myomiRs (miR‐1, miR‐133a, miR‐208, miR‐499) and miR‐214, can have a role in the right ventricle in the development of PAH. To mimic PAH, male Wistar rats were injected with monocrotaline (MCT, 60 mg/kg, s.c.); control group received vehicle. MCT rats were divided into two groups, based on the clinical presentation: MCT group terminated 4 weeks after MCT administration and prematurely terminated group (ptMCT) displaying signs of terminal disease. Myocardial damage genes and candidate microRNAs expressions were determined by RT‐qPCR. Reduced blood oxygen saturation, breathing disturbances, RV enlargement as well as elevated levels of markers of myocardial damage confirmed PH in MCT animals and were more pronounced in ptMCT. MyomiRs (miR‐1/miR‐133a/miR‐208a/miR‐499) were decreased and the expression of miR‐214 was increased only in ptMCT group (*P* < 0.05). The myomiRs negatively correlated with Fulton index as a measure of RV hypertrophy in MCT group (*P* < 0.05), whereas miR‐214 showed a positive correlation (*P* < 0.05). We conclude that the expression of determined microRNAs mirrored the disease severity and targeting their pathways might represent potential future therapeutic approach in PAH.

## 1 INTRODUCTION

Pulmonary arterial hypertension (PAH) is a rare and severe disease, defined by an elevation in mean pulmonary arterial pressure^1^ caused by increased vasoconstriction, vascular remodelling, fibrosis and stiffening of the pulmonary vasculature. The disease progresses into elevated right ventricular (RV) afterload promoting RV failure, the leading cause of death for the patients with PAH.^2^ Notwithstanding that medical interventions decrease pulmonary vascular resistance, deterioration in RV function occurs in a certain number of patients, which has a strong prognostic value in PAH.^3^ Therefore, RV‐targeted therapy is needed. Multifactorial background and epigenetic alterations might also markedly contribute to PAH progression starting to be explored for therapies mostly in animal models.^4^ In rats, administration of monocrotaline induces a reliable model of experimental pulmonary hypertension (PH) which mimics a number of features of human PAH.^5^, ^6^, ^7^, ^8^

MicroRNAs (miRNAs) are small non‐coding RNA molecules (around 22 nucleotides long) that regulate gene expression on post‐transcriptional level, by mRNA degradation or translational repression^9^ and various miRNAs have been suggested to be involved in the pathophysiology of PAH.^10^, ^11^ Importantly, alterations in muscle‐specific microRNAs (myomiRs), that is miR‐1, miR‐133a, miR‐208 and miR‐499, have been found in diverse cardiac injuries^12^, ^13^ and, recently, myomiRs have been unveiled to be sensitive markers of sudden death from myocardial infarction.^14^ Thus, their role in RV pathology resulting from PAH can be anticipated.

MiR‐1 is abundantly expressed in the skeletal muscle and the heart and is a key regulator in differentiation and proliferation of muscular cells.^13^ MiR‐1 also plays a crucial role during cardiogenesis and regulates cardiac conduction.^15^ Its increased levels have been detected in patients with coronary arterial disease and in rat hearts after myocardial infarction.^16^ On the contrary, decreased levels were observed in patients with atrial fibrillation^17^ and in rats with left ventricular hypertrophy.^18^

Closely related to miR‐1, miR‐133 has two isoforms, miR‐133a and miR‐133b. MiR‐133a is highly expressed in the heart and also plays a role in cardiogenesis and cardiac conductance.^13^ It has antiapoptotic properties in cardiomyocytes^19^ and represses myocardial fibrosis.^20^ Similarly to miR‐1, its expression was also found to be decreased in cardiac hypertrophy.^21^

Another heart‐enriched microRNAs belong to the miR‐208 family, which consists of miR‐208a, encoded by α‐myosin heavy chain (MHC) gene—Myh6; miR‐208b, co‐expressed with β‐MHC gene—Myh7; and miR‐499, encoded in the Myh7b gene. In adult rodents, miR‐208a is expressed exclusively in the cardiac muscle, thus cardio‐specific, whereas miR‐499 is relatively highly expressed in the heart and miR‐208b is absent in this organ under normal conditions. α‐MHC is also the predominant form of MHC, accounting for more than 90% of total expression.^22^ However, in larger mammals and human beings, the major cardiac isoform is β‐MHC.^23^ During pathological hypertrophy, the distribution of myosin isoform changes with induction of β‐MHC at the expense of α‐MHC.^24^ MiR‐208a regulates the expression of Myh7b/miR‐499 and is also needed for the up‐regulation of Myh7/miR‐208b and β‐MHC in pathological setting. This microRNA is therefore considered to govern myofiber diversification, stress responsiveness of the heart and cardiac remodelling^22^, ^25^ and it is also important for proper cardiac conduction.^26^ MiR‐499, seeming to be a downstream mediator of miR‐208a,^22^ has antiapoptotic and proliferative properties during the late stages of cardiac differentiation.^27^

Even though miR‐214 was initially associated with cellular death and is dysregulated in various malignancies, it is also expressed in cardiac tissue having cardiac pathology‐related influence, influencing tissue remodelling, ischaemic conditions, etc^28^ This microRNA was investigated in connection with cardiac hypertrophy and fibrosis; nevertheless, the studies suggest controversial conclusions, because some indicate that miR‐214 has pro‐hypertrophic^29^ and pro‐fibrotic^30^ properties, whereas others showed its protective effects in these aspects.^31^, ^32^

Based on a close relation of the above‐mentioned miRNAs to cardiac injury and remodelling, we suppose that these microRNAs could also have an impact on the overwhelmed right ventricle in experimental PH and they could possibly help clarify pathological mechanisms occurring in this organ.

## 2 MATERIALS AND METHODS

### 2.1 Animal experiment design

In the experiment, 12‐week‐old male Wistar rats weighing 235‐300 g (Department of Toxicology and Laboratory Animals breeding, Dobrá Voda, Slovak Republic) were randomized into two groups, depending on the treatment (either saline—control group or monocrotaline—MCT group). Monocrotaline was administered subcutaneously at the dose of 60 mg/kg in MCT group^7^ whereas the control group received a corresponding volume of saline. Rats were kept under standard conditions, with access to food and water ad libitum. The rats were planned to be killed after 4 weeks since the treatment administration; however, in certain animals from the MCT group, the terminal stage of PH developed more rapidly and they had to be killed prematurely, since their survival till the 28th day after MCT administration was not expected. These prematurely terminated MCT rats (ptMCT) were observed and selected by two independent examiners, the necessary criteria being loss of more than 10 g in bodyweight during 24 hours, estimated profound dyspnoea (confirmed by breath rate measurement, see Vital functions measurements), decreased activity and apathy and bristled fur. In our experience, all of these signs indicate a potential death in 24 hours. Therefore, ptMCT group represents the terminal stages of the progression of PH. The animals in the control and surviving MCT group were killed 4 weeks after the treatment, as planned. All experimental procedures that involved the use of experimental animals were approved by the Ethics Committee of the Faculty of Pharmacy, Comenius University in Bratislava, Slovak Republic; and by the State Veterinary and Food Administration of the Slovak Republic. These investigations were also performed in accordance with NIH Guide for the Care and Use of Laboratory Animals: Eight Edition (2010) published by the US Committee for the Update of the Guide for the Care and Use of Laboratory Animals; National Research Council, the EU adopted Directive 2010/63/EU of the European Parliament and of the Council on the protection of animals used for experimental and other scientific purposes and the Slovak law regulating animal experiments.

### 2.2 Vital functions measurements

For haemoglobin oxygen saturation, heart and breath rate measurement, a pulse oximeter (MouseOx Plus; Starr Life Sciences, Oakmont, PA, USA) was used 24 hours before sacrifice,^6^ or, in case of ptMCT group, shortly before sacrifice. A sensor collar of appropriate size was placed around the neck of conscious rats, and the vital functions of the animals were recorded and analysed accordingly to the manufacturer's instructions.

### 2.3 Sample collection

Lungs and heart ventricles were harvested, blotted dry and weighted. The ratio of RV mass to left ventricular mass plus interventricular septum (Fulton index) was used as a measure of RV hypertrophy.^8^ Tissue samples of right ventricles were frozen in liquid nitrogen and stored at −80°C until further processing.

### 2.4 Messenger RNA and microRNA tissue expression measurement

Both mRNA expression of cardiac failure‐related indicators, such as brain natriuretic peptide (BNP)‐encoding gene (Nppb),^33^ cardiac muscle myosin heavy chains (α‐ and β‐MHC) encoding genes (Myh6 and Myh7)^12^ and microRNA expression in RV samples, were determined by quantitative real‐time polymerase chain reaction (RT‐qPCR). Total RNA was isolated by phenol/chloroform extraction method using TRI reagent (Sigma‐Aldrich, Saint‐Louis, MO, USA). The quality of the isolated RNA was verified by 2% agarose gel electrophoresis and quantified by microspectrophotometry using NanoDrop ND‐1000 (Thermo Fisher Scientific, Waltham, MA, USA).

For mRNA measurements, reverse transcription was performed using High Capacity cDNA Reverse Transcription Kit with RNAse inhibitor (Thermo Fisher Scientific) with 2 μg of total RNA. SYBR^™^ Select Master Mix (Thermo Fisher Scientific) was utilized for qPCRs. Messenger RNA‐specific primers (Sigma‐Aldrich) were designed in Primer‐BLAST^34^ with measures against genomic DNA detection and were verified to yield a single PCR product with the correct molecular weight and amplicon length. List of primers with their sequences is shown in Table 1. Results were normalized to the geometric mean of expressions of two pre‐verified endogenous reference genes—beta‐2‐microglobulin and hypoxanthine phosphoribosyltransferase 1.

**TABLE 1:** Primer sequences for RT‐qPCR

Gene symbol	RefSeq access. no.	Primer sequences (5′ → 3′)	PCR product size
B2m	NM_012512.1	Forward: ATG GAG CTC TGA ATC ATC TGG Reverse: AGA AGA TGG TGT GCT CAT TGC	105
Hprt1	NM_012583.2	Forward: CAG CTT CCT CCT CAG ACC GCT TT Reverse: TCA CTA ATC ACG ACG CTG GGA CTG	150
Myh6	NM_017239.2	Forward: GCC CTT TGA CAT CCG CAC AGA GT Reverse: TCT GCT GCA TCA CCT GGT CCT CC	152
Myh7	NM_017240.1	Forward: GCG GAC ATT GCC GAG TCC CAG Reverse: GCT CCA GGT CTC AGG GCT TCA CA	133
Nppb	NM_031545.1	Forward: GAC CGG ATC GGC GCA GTC AGT Reverse: GGA GTC TGC AGC CAG GAG GTC T	78

Note

Abbreviations: B2m, beta‐2‐microglobulin; Hprt1, hypoxanthine phosphoribosyltransferase 1; Myh6, myosin heavy‐chain 6; Myh7, myosin heavy‐chain 7; Nppb, natriuretic peptide B.

For microRNA measurements, reverse transcription was performed using TaqMan MicroRNA Reverse Transcription Kit (Applied Biosystems, Foster City, CA, USA) in a multiplex assay of all analysed microRNAs according to the manufacturer's instructions. TaqMan 2× Universal PCR Master Mix (Applied Biosystems) was utilized for qPCRs with primers from individual TaqMan microRNA Assays (Applied Biosystems) of analysed microRNAs (Table 2). Results were normalized to the expression of endogenous reference, U6 small nuclear RNA.

**TABLE 2:** Mature sequences of rat cardiac microRNAs (seed sequences are underlined, source mirBase.org) and control U6 snRNA

microRNA name	Nomenclature of mature form	Sequence
miR‐1	rno‐miR‐1‐3p	5′‐UGGA AUG UAA AGA AGU GUG UAU‐3′
miR‐133a	rno‐miR‐133a‐3p	5′‐UUUG GUC CCC UUC AAC CAG CUG‐3′
miR‐208a	rno‐miR‐208a‐3p	5′‐AUAA GAC GAG CAA AAA GC‐3′
miR‐214	rno‐miR‐214‐3p	5′‐ACAG CAG GCA CAG ACA GGC AG‐3
miR‐499	rno‐miR‐499‐5p	5′‐UUAA GAC UUG CAG UGA UGU UU‐3′
U6	U6 snRNA	5′‐GTGCTCGCTTCGGCAGCACATATAC TAAAATTGGAACGATACAGAGAAGAT TAGCATGGCCCCTGCGCAAGGATGAC ACGCAAATTCGTGAAGCGTTCCATATT TT‐3′

All qPCRs of both mRNAs and microRNAs were performed on StepOnePlus^™^ Real‐Time PCR System (Thermo Fisher Scientific). Mean PCR efficiency estimates per amplicon and quantification cycle (Cq) values per sample were determined with LinRegPCR software (version 2018.0).^35^ Finally, Pfaffl‐like efficiency corrected relative expressions were calculated^36^ and calibrated to their applicable control group.

### 2.5 Statistical analysis

Data are presented as mean ± SEM. Data distribution was determined by Shapiro‐Wilk normality test. One‐way unbalanced ANOVA for single‐factor analysis (ie intensity of MCT effect) with Tukey's HSD post hoc test was selected for normally distributed results; otherwise, Kruskal‐Wallis test was chosen, with pairwise Wilcox post hoc test adjusted for multiple comparison by Benjamini‐Hochberg procedure. Results were considered statistically significant when two‐tailed *P* < 0.05. Pearson correlation coefficient in MCT group was calculated for bivariate normally distributed pairs of variables, otherwise Spearman correlation coefficient was computed. Correlations with |*r*| > 0.60 for sample size N = 20 in MCT group have *P* < 0.05 and statistical power > 0.83 and were considered statistically significant. All calculations were performed in GraphPad Prism 5 (GraphPad Software, San Diego, CA, USA) or in R, version 3.5.2.^37^

## 3 RESULTS

In rats following MCT administration, the development of PH was demonstrated by increased RV mass (including Fulton index), heart rate, lung weight and depressed oxygen saturation (Table 3). With exception of heart rate and oxygen saturation, all relevant characteristics were significantly elevated in ptMCT rats when compared to surviving MCT animals. Additionally, ptMCT rats exhibited also markedly elevated breath rate.

**TABLE 3:** Basic characteristics of experimental groups

	Control	MCT	ptMCT
Bodyweight (g)	381 ± 5	338 ± 8*	308 ± 8*^#^
Heart mass (g)	1.10 ± 0.03	1.14 ± 0.04	1.37 ± 0.05*^#^
Heart mass to bodyweight ratio (mg/g)	2.89 ± 0.05	3.38 ± 0.18	4.44 ± 0.11*^#^
Right ventricle mass (g)	0.22 ± 0.01	0.30 ± 0.02*	0.44 ± 0.02*^#^
Right ventricle mass to BW ratio (mg/g)	0.59 ± 0.01	0.90 ± 0.07*	1.44 ± 0.06*^#^
Left ventricle mass (g)	0.78 ± 0.02	0.70 ± 0.02*	0.73 ± 0.03
Left ventricle mass to BW ratio (mg/g)	2.03 ± 0.03	2.07 ± 0.06	2.37 ± 0.05*^#^
Fulton index	0.29 ± 0.01	0.43 ± 0.02*	0.61 ± 0.03*^#^
Lung mass (g)	1.54 ± 0.08	2.06 ± 0.10*	2.78 ± 0.12*^#^
Lung mass to BW ratio	4.03 ± 0.19	6.19 ± 0.36*	9.08 ± 0.49*^#^
Oxygen saturation (%)	94.3 ± 0.5	92.0 ± 0.5*	92.0 ± 0.7*
Heart rate (bpm)	399 ± 6	431 ± 9*	451 ± 8*
Breath rate (brpm)	102 ± 2	109 ± 4	157 ± 9*^#^

Note

Bodyweight (BW), absolute and relative mass of organs, Fulton index (right ventricular mass/left ventricular plus septum mass), haemoglobin oxygen saturation, heart and breath rate of the rats; n(control) = 17, n(MCT) = 20, n(ptMCT) = 10; expressed as mean ± SEM, *P < 0.05 vs control group, ^#^ *P *< 0.05 vs MCT.

Abbreviations: bpm, beats per minute; brpm, breaths per minute; MCT, monocrotaline group; ptMCT, prematurely terminated MCT group.

### 3.1 Changes in the gene expression of RV impairment markers

**FIGURE 1:**
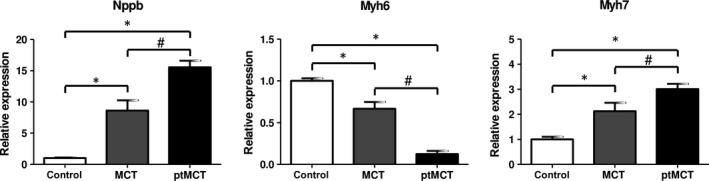
Relative gene expression of Nppb, Myh6 and Myh7 in the right ventricle; n(control) = 17, n(MCT) = 19, n(ptMCT) = 9; expressed as mean ± SEM, *P < 0.05 vs control group, ^#^ *P* < 0.05 vs MCT. MCT, monocrotaline group; Myh6, myosin heavy‐chain 6; Myh7, myosin heavy‐chain 7; Nppb, natriuretic peptide B; ptMCT, prematurely terminated MCT group

We noted a significant eightfold increase in the expression of BNP encoding gene (Nppb) in MCT group and a 15‐fold increase of Nppb in ptMCT when compared to control group (Figure 1). Gene expression of Myh6 was significantly decreased in MCT group (by 33%); this was aggravated in ptMCT group (by 88%) vs the control group. In contrast, the Myh7 gene was up‐regulated according to a similar pattern, that is with more considerable change in ptMCT (Figure 1).

### 3.2 Dysregulated expression of myomiRs and miR‐214 in right ventricle

**FIGURE 2:**
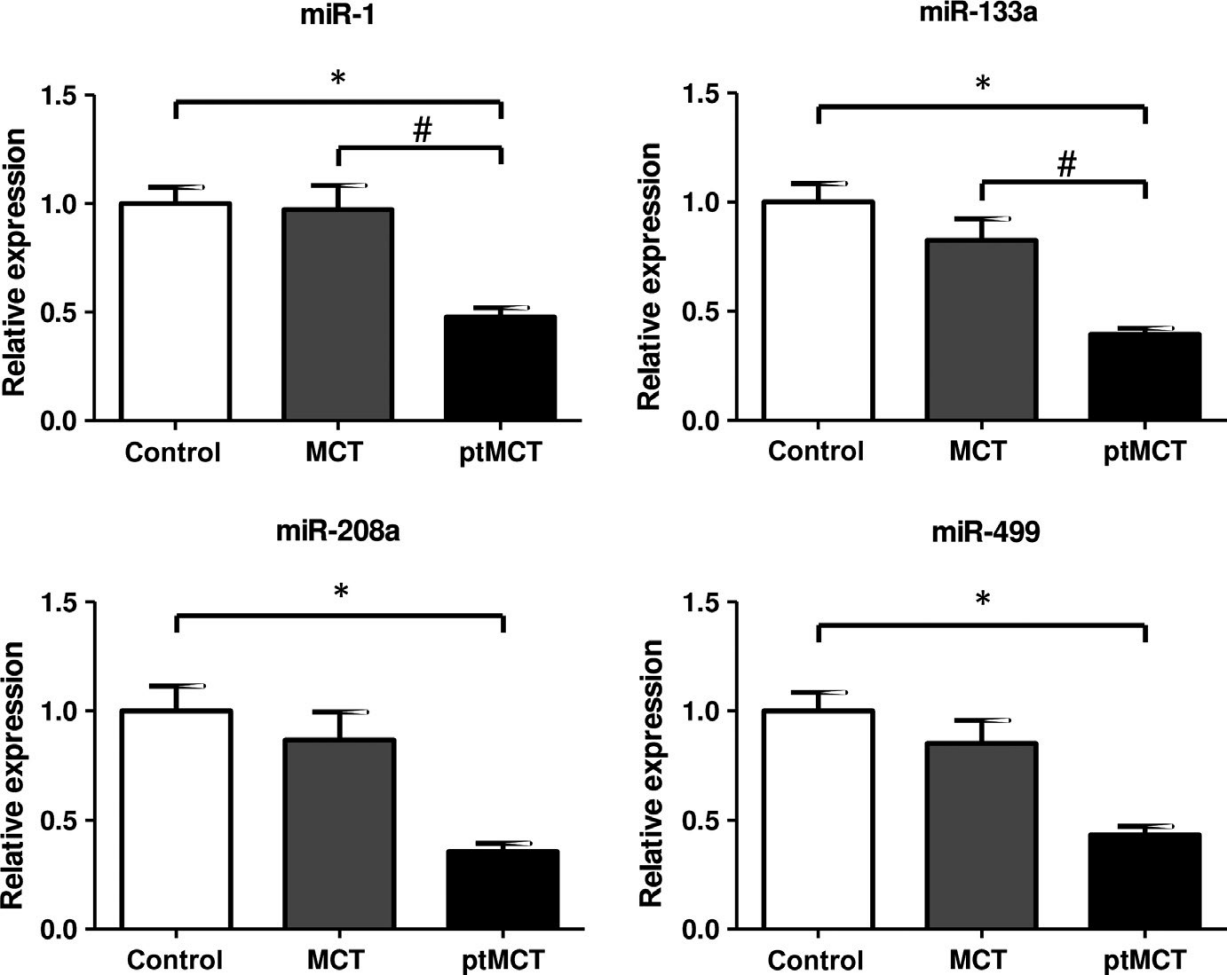
Relative microRNA expression of miR‐1, miR‐133a, miR‐208a and miR‐499 in the right ventricle; n(control) = 17, n(MCT) = 20, n(ptMCT) = 10; expressed as mean ± SEM, *P < 0.05 vs control group, ^#^ *P *< 0.05 vs MCT. MCT, monocrotaline group; ptMCT, prematurely terminated MCT group

**FIGURE 3:**
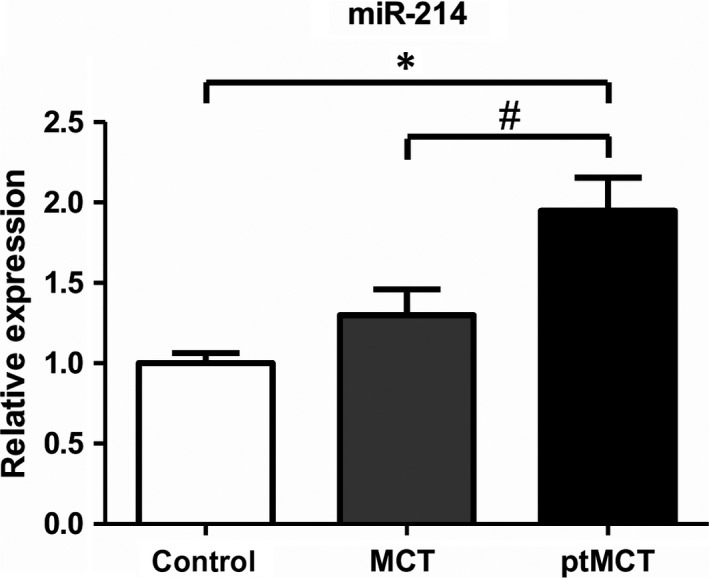
Relative microRNA expression of miR‐214 in the right ventricle; n(control) = 17, n(MCT) = 20, n(ptMCT) = 10; expressed as mean ± SEM, *P < 0.05 vs control group, ^#^ *P *< 0.05 vs MCT. MCT, monocrotaline group; ptMCT, prematurely terminated MCT group

We found significantly suppressed RV tissue levels of all tested myomiRs exclusively in the ptMCT group (when compared to control group, in case of miR‐1 and miR‐133a also compared to MCT, see Figure 2) whereas these were stable in MCT. Expression of miR‐214 was unaltered in MCT group whereas significant up‐regulation occurred in ptMCT group (by 95%) when compared to control group (Figure 3).

### 3.3 Expression of microRNAs significantly correlates with Fulton index

**FIGURE 4:**
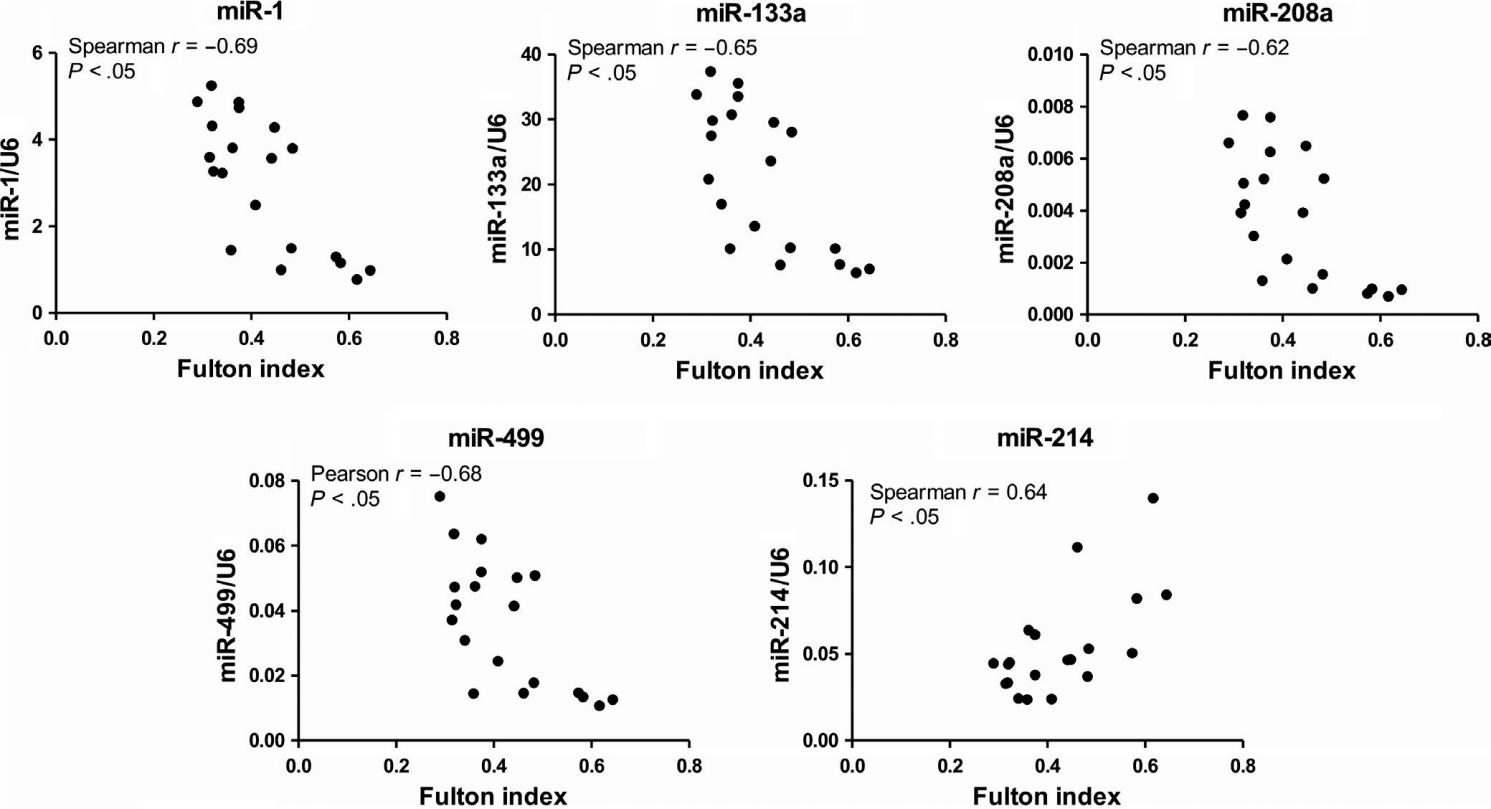
Correlation between relative microRNA expression of miR‐1, miR‐133a, miR‐208a, miR‐214 and miR‐499 (when normalized to reference U6 small nuclear RNA) in the right ventricle and Fulton index (right ventricular mass/left ventricular plus septum mass) in MCT group (n = 20; *P* < 0.05)

Further analyses showed a significant negative correlation between the expression of miR‐1, miR‐133a, miR‐208a and miR‐499 and Fulton index in the MCT group (Figure 4). In contrary, there was also a significant positive correlation between miR‐214 and Fulton index.

## 4 DISCUSSION

In this study, we observed significant alterations of candidate microRNAs in the right ventricles of monocrotaline‐induced model of PH in rats in line with disease severity. The right ventricle is an essential point of interest in PAH as it is the major determinant of functional state and prognosis of the disease.^38^ We focused on heart‐relevant microRNAs, that is miR‐1, miR‐133a, miR‐208a, miR‐499 and miR‐214 due to their high expression in the myocardium and their vital roles in cardiac development and function, and an increasing evidence about their roles in cardiovascular pathologies.

We discriminated between two groups of rats suffering from MCT‐induced PH according to their disease progression. As expected, ptMCT group exhibited more pronounced signs of PH development and target organ damage compared to surviving MCT group, in line with reports that clinical manifestation correlates with the stage of experimental PH development.^39^ In addition, ptMCT exhibited altered tissue expression of cardiac damage‐related genes. First, RV expression of BNP encoding gene (Nppb) was enhanced in MCT and this up‐regulation was more pronounced in ptMCT rats. This is in agreement with the known fact that BNP levels are increasing during ventricular failure progression.^40^ It is also well reported that MHCs are changing during myocardial remodelling and dysfunction, and hence, alterations in Myh6 and Myh7 genes, in particular in ptMCT group, were expected.^41^ Taken together, gross clinical examination unveiled the terminal stage of PH reliably and was accompanied by foetal gene re‐expression (Nppb, Myh7), an event correlating with experimental cardiac failure.^12^

MyomiRs are intensively studied in diverse models of cardiac diseases,^13^ including those of PH.^39^, ^42^, ^43^ Although MCT administration is the most common in PH research, the data on myomiRs in right ventricle of this particular model are from scarce to non‐existent, with the exception of miR‐208.^39^ Here, we describe a concomitant suppression of muscle‐related microRNAs, including the cardiac‐specific miR‐208a, exclusively in the terminal stage of the disease. Moreover, this finding is underlined with a significant, negative correlation of myomiR levels with Fulton index, an indicator of RV hypertrophy progression, in surviving MCT rats with less severe disease. As translational silencing of mRNAs is the dominant mechanism of microRNAs,^9^ the lack of myomiR expression, particularly in ptMCT, could be interpreted as a loss of inhibitory control of myomiR network on target mRNAs potentially contributing to RV deterioration. This requires further confirmation; nevertheless, myomiRs probably do not govern the initial phases of RV remodelling in MCT‐induced PH, but their suppressions might contribute to the worsening of the RV deterioration at the terminal stage.

Particularly, we have found depressed levels of miR‐208 family members (miR‐208a and miR‐499, resp.). MiR‐208a, as the only cardiac‐specific myomiR, is usually found to be dysregulated in various cardiac pathological situations, like myocardial infarction and dilated cardiomyopathy.^44^, ^45^ Interestingly, decreased miR‐208 expression was reported in the stage of compensated RV hypertrophy in the MCT‐induced model of PH, with a further aggravation in decompensated RV hypertrophy.^39^ This progressive decrease may cause, for instance, a repression of transcription factor Mef2 via MED13/NoR1 complex, marking the transition from compensated RV hypertrophy to the decompensated state, because Mef2 has been implicated in regulation of various metabolic, contractile and angiogenic genes.^39^ Importantly, miR‐208 family members control also the regulation of MHCs distribution participating in an adaptation of adult cardiac gene expression to pathological stimuli. Their cistrons are located in the introns of the cardiac muscle α‐ and β‐MHC‐encoding genes (Myh6, Myh7, resp.) responsible for cardiac contractility and are excised from the pre‐mRNA.^46^ In particular, miR‐208a encoded by the Myh6 gene is the dominant regulatory microRNA in this family^22^ and plays a crucial role in the cardiac stress response.^12^ Cardiac stress stimuli were often associated with decreased expression of α‐MHC mRNA (Myh6) and/or increased β‐MHC mRNA (Myh7) expression in both rodents and human beings.^24^ This is usually followed by a decrease in miR‐208 expression, which, according to some authors,^25^ should indirectly lead to down‐regulation of β‐MHC expression. This seemingly contradictory finding was later partially explained by the knowledge^47^ that some microRNAs are capable of functioning properly up to a certain threshold. Thus, even low levels of miR‐208 are perhaps able to sustain increase in β‐MHC expression. This was also seen in other experimental cardiomyopathies.^12^ Interestingly, miR‐208a/b and miR‐499 quantities were increased in left ventricular heart failure,^48^ suggesting a differential expression pattern in various cardiac compartments.^49^

MiR‐1 regulates the expression of genes relevant in cardiac conduction and contraction.^13^ The down‐regulation we observed was also noted in the murine model of pulmonary artery constriction.^43^ Contradictory to our observation, Reddy and colleagues reported the down‐regulation of miR‐1 in RV as soon as 2 days after pulmonary artery constriction which they described as early compensated hypertrophy. This down‐regulation persisted until progression to decompensated cardiac hypertrophy and failure, in line with our findings.^43^ Altered miR‐1 levels lead to conductance defects of heart.^50^, ^51^ In both human PAH and MCT‐induced PH, a proarrhythmic state is a vital contributor to morbidity and mortality.^52^, ^53^ The decrease in miR‐1 levels could contribute to this condition.

With regard to miR‐133a that is also a regulator of cardiac electric remodelling and exhibits antifibrotic and antihypertrophic effects in heart,^54^, ^55^ we observed a marked decrease only in ptMCT. Drake and colleagues found down‐regulation of miR‐133a only in models of RV failure (using Sugen5416/hypoxia and pulmonary artery banding with a Cu^2+^‐depleted diet), whereas in the models of compensated RV hypertrophy (hypoxia and pulmonary artery banding), they observed no significant changes.^42^ This is perfectly in accordance with our results, that is the more pronounced RV damage, the lower the miR‐133a expression.

So far, miR‐214 has been linked to different effects in studies concerning cardiac hypertrophy and fibrosis.^29^, ^30^, ^31^, ^32^ Similarly to our findings in ptMCT rats, miR‐214 was found to be up‐regulated in the right ventricle of the Sugen5416/hypoxia PH model^56^ that is considered a model of severe PH with RV failure.^42^ Reddy and colleagues also reported increased miR‐214 expression in pulmonary artery banding model of RV hypertrophy and failure and associated this microRNA with myocyte survival and antiapoptotic properties.^43^ MiR‐214 null male mice exposed to Sugen5416 and hypoxia had more pronounced RV hypertrophy when compared to their respective wild‐type controls. Interestingly, this was not associated with elevation in RV systolic pressure.^56^ This would suggest that miR‐214 has a direct, pressure independent, antihypertrophic effect on the right ventricle. However, it is rather contradictory to our finding of significantly positive correlation between miR‐214 and the measure of RV hypertrophy, that is Fulton index, in surviving MCT rats and to the observation that miR‐214 elevation was significantly present only in ptMCTs. Alternatively, one could interpret the finding of enhanced miR‐214 as crucial contribution to RV damage because this microRNA plays an essential role in the inhibition of cardiac angiogenesis.^57^

There are certain limitations in the present study. MCT model—even though widely used, as other animal models, does not entirely reflect the human pathophysiology. Also, when translating findings concerning miR‐208a to the clinical setting, we need to keep in mind that Myh6/miR‐208a is the predominant myosin/myomiR isoform in the hearts of rodents whereas larger mammals express mainly Myh7/miR‐208b.^12^

To sum up, alterations in RV tissue levels of candidate microRNAs reflected the worsened clinical status of rats and the more pronounced molecular indicators of cardiac damage in MCT‐induced PH. We showed decreased RV levels of cardio‐specific miR‐208a as well as other myomiRs (miR‐1, miR‐133a and miR‐499), whereas the expression of cardiac damage‐related miR‐214 was enhanced with disease severity. Further studies should focus on their particular downstream targets in preclinical research and drug development. In conclusion, our data support the validity of concept of influential microRNAs in PH‐related RV damage inspiring their further investigation as potential aid for differential diagnosis of human disease.

## 5 CLINICAL IMPLICATIONS

In this study, we showed the decreased levels of myomiRs and increased expression of miR‐214 at the terminal stage of experimental PH. As these microRNAs were demonstrated to be fundamental regulators also in human cardiac pathology,^14^, ^57^ they might be in the spotlight of clinical research when searching for candidate molecules responsible for sudden worsening of RV performance in human PAH patients.

## CONFLICT OF INTEREST

The authors confirm that there are no conflicts of interest.

## AUTHOR CONTRIBUTIONS

ZK, KLZ and JV performed the research; ZK, LBP, GD and EM analysed the data and devised the methodology; ZK, JK and PK wrote the paper; LP revised the manuscript; JK and PK designed the research study and supervised the work.

## Data Availability

The data that support the findings of this study are available from the corresponding author upon reasonable request.
